# Global identification of *LIM* genes in response to different heat stress regimes in *Lactuca sativa*

**DOI:** 10.1186/s12870-024-05466-x

**Published:** 2024-08-06

**Authors:** Taehoon Kim, Andrew Egesa, Claire Qin, Hannah Mather, Germán Sandoya, Kevin Begcy

**Affiliations:** 1 Environmental Horticulture Department, University of Florida, Gainesville, FL 32611 USA; 2 Student Science Training Program, University of Florida, Gainesville, FL 32611 USA; 3 Horticultural Science Department, University of Florida, Gainesville, FL 32611 USA; 4 Everglades Research and Education Center, Horticultural Sciences Department, University of Florida IFAS, Belle Glade, FL 33430 USA

**Keywords:** Thermoresilience, Phylogenetics, Climate change, Gene expression

## Abstract

**Background:**

*LIM* (Lineage-11 (LIN-11), Insulin-1 (ISL-1), and Mechanotransduction-3 (MEC-3)) genes belong to a family that hold ubiquitous properties contributing to organ, seed, and pollen development as well as developmental and cellular responses to biotic and abiotic stresses. Lettuce (*Lactuca sativa*) is a highly consumed vegetable crop susceptible heat stress. High temperatures limit lettuce’s overall yield, quality and marketability. Lettuce *LIM* genes have not been identified and their role in response to high temperatures is not known. Aiming to identify potential new targets for thermoresilience, we searched for LIM genes in lettuce and compared them with orthologous of several dicotyledons and monocotyledons plant species.

**Results:**

We identified fourteen lettuce *LIM* genes distributed into eight different subgroups using a genome-wide analysis strategy. Three belonging to *DAR* (DA means “large” in Chinese) class I, two *DAR* class II, one in the *WLIM1*, two in the *WLIM2*, one in the *PLIM1*, two in the *PLIM2* class, one *ßLIM* and two *δLIMs*. No DAR-like were identified in any of the species analyzed including lettuce. Interestingly, unlike other gene families in lettuce which underwent large genome tandem duplications, *LIM* genes did not increase in number compared to other plant species. The response to heat stress induced a dynamic transcriptional response on *LsLIM* genes. All heat stress regimes, including night stress, day stress and day and night stress were largely responsible for changes in *LIM* transcriptional expression.

**Conclusions:**

Our global analysis at the genome level provides a detailed identification of *LIM* genes in lettuce and other dicotyledonous and monocotyledonous plant species. Gene structure, physical and chemical properties as well as chromosomal location and Cis-regulatory element analysis together with our gene expression analysis under different temperature regimes identified *LsWLIM1*, *LsWLIM2b*, *LsDAR3* and *LsDAR5* as candidate genes that could be used by breeding programs aiming to produce lettuce varieties able to withstand high temperatures.

**Supplementary Information:**

The online version contains supplementary material available at 10.1186/s12870-024-05466-x.

## Background

Recent climate data has shown an increase in global temperature at an average rate of 0.08 degrees Celsius per decade since 1880 [1]. However, a more drastic increase has been observed since 1981, when the average rate of increase was calculated at 0.18 °C; more than twice from previous decades [1]. As global temperatures are on the rise, crop production is threatened by the current climate change scenario represented by variable increase of high temperatures [2–6]. These temperature regimes vary in duration and intensity. Major yield losses due to high temperatures have been reported for many crop species [2–6]. Among the plant species that are severely affected by high temperatures is lettuce (*Lactuca sativa*). Lettuce production is largely based on field and control environment conditions. High temperature during lettuce field production results in less yield and the appearance of disorders including tip-burn, and bolting and other not desirable traits that limit its marketability [7]. Most studies investigating lettuce in response to high temperatures have been focused on the effects on crop physiology, yield, and quality [8–12]. Lettuce that tolerates higher temperatures are prone to less transpiration and produce more sugars in response to warmer environment [8, 10]. At the genomic level, many gene families have shown to be stress responsive. In plants, many gene families are heat-stress responsive [13]. However, the role of *LIM* genes in response to environmental stresses has not been characterized specially in response to increased temperatures.

LIM proteins (Lineage-11 (LIN-11), insulin-1 (ISL-1), and mechanotransduction-3 (MEC-3)) belong to a small gene family class found in plant and animals [14–16]. In plants, LIM proteins have been shown to be involved in a variety of processes during vegetative and reproductive development (See review [14]). LIM proteins have been proposed to protect against biotic stresses by regulating salicylic acid (SA), jasmonic acid (JA) and abscisic acid (ABA) under stress conditions [17]. LIM proteins contain one or several (up to five) double zinc finger motifs, known as LIM domains, which function by mediating protein–protein interactions. LIM domains have been found in a wide variety of eukaryotic proteins of diverse functions [16]. LIM domain-containing transcription factors without the homeodomain have also been described. Specifically, the LIM-only protein LMO2 was found to act as a bridging molecule in assembly of the erythroid DNA-binding complex, while other LIM-domain-containing proteins such as LIM kinases are known to participate in regulation of actin dynamics through phosphorylation of cofilin [18]. In contrast, plants possess two distinct sets of LIM proteins, one that is plant–specific and has been partially functional characterized. Another, cysteine rich protein (CRP)-like that comprises CRPs exhibiting the same overall structure found in animals (i.e., two very similar LIM domains separated by an ≈ 50 amino acid–long inter LIM domain and a relatively short and variable C–terminal domain).

Here, we describe a global identification and analysis of *LIM* genes under a diverse set of temperature regimes. A comprehensive description of each LIM subclass including gene structure, physical and chemical properties as well as chromosomal location and cis-regulatory element analysis is provided. This study provides a new set of candidate genes that can be used for plant breeders to generate thermotolerant lettuce germplasm.

## Results

### Genome-wide identification and phylogenetic analysis of *LIM* genes in lettuce and other plant species

With the aim of identifying potential new targets for thermoresilience, we searched for *LIM* genes in several dicotyledons and monocotyledons plant species. In the model plant *Arabidopsis thaliana*, thirteen LIM domain-containing genes have been previously identified: Seven *DA1/DAR* genes (containing one LIM domain) and six *2LIM* or CRP-like genes (containing two LIM domains) [19, 20]. We used these *Arabidopsis* LIM proteins as query sequences and performed an automated local BLASTP search to identify LIM family members of lettuce (*Lactuca sativa*), sunflower (*Helianthus annuus)*, tobacco (*Nicotiana tabacum*), *Brachypodium distachyon*, barley (*Hordeum vulgare*), rice (*Oryza sativa*), maize (*Zea mays*), sorghum (*Sorghum* bicolor), and foxtail millet (*Setaria italica*) (Additional File 1: Table S1). Only LIM proteins that we confirmed to possess the expected numbers of LIM domains or DA1-like domains were retained for further analysis. In lettuce, we identified five DARs gene and nine *2LIM* genes.

**Fig. 1:**
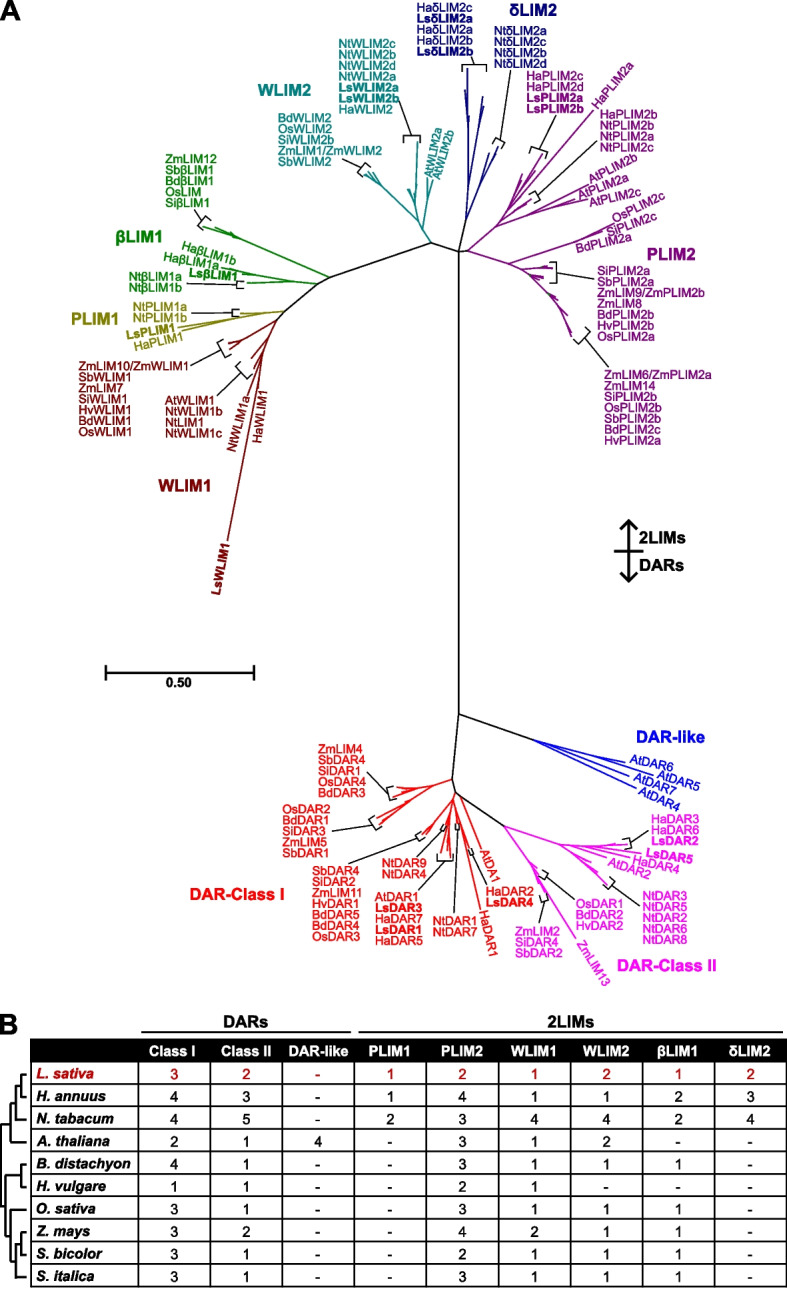
Phylogenetic analysis of *LIM* genes in different plant species. **A** The amino acid sequences of LIM proteins from *A. thaliana* (At), *B. distachyon* (Bd), *H. annuus* (Ha), *H. vulgare* (Hv), *L. sativa* (Ls), *N. tabacum* (Nt), *O. sativa* (Os), *S. italica* (Si), *S. bicolor* (Sb), and *Z. mays* (Zm) were aligned and used to construct a phylogenetic tree was using the Maximum Likelihood method and Whelan and Goldman (WAG) model [21] with 1000 Bootstrap replications. Genes belonging to different classes were differently colored. Maroon, WLIM1; olive, PLIM1; green, βLIM1; teal, WLIM2; navy, δLIM2; purple, PLIM2; red, DAR-class I; fuchsia, DAR-class II; blue, DAR-like. **B** Number of *LIM* genes identified in diverse plant species. Phylogenetic relationship among different plant species was constructed based on Zhao et al*.* [22]

After our genome-wide identification of *LIM* genes in lettuce, sunflower, tobacco, *Arabidopsis*, brachypodium, barley, rice, maize, sorghum, and foxtail millet, we performed a phylogenetic analysis aiming to elucidate the evolutionary relationship of *LIM* genes within the dicotyledons and monocotyledons species used in this study (Fig. 1A; Additional Fig. S1). Our results separate the identified *LIM* genes into nine subgroups. The *WLIM1* subgroup contains members of all the ten plant species. Interestingly, most species only have one *WLIM1* gene. However, maize has two and tobacco four (Fig. 1B). Evolutionarily, dicotyledons and monocotyledons *WLIM1* genes were grouped in two clear separated clades. Within the dicotyledons, *WLIM1a* genes from lettuce, sunflower, and tobacco clustered together. A different cluster was formed by the other three *WLIM1* genes from tobacco and the Arabidopsis ortholog (Fig. 1A). Similarly, *WLIM2* genes clustered based on their monocotyledonous or dicotyledonous origin. However, *Arabidopsis WLIM2* diverged from the other dicotyledonous plant species analyzed (Fig. 1A).

*δLIM2* forms an interesting subgroup with only genes from lettuce, sunflower, and tobacco. Remarkably, our analysis shows that tobacco *δLIM2* genes diverged earlier form lettuce and sunflower *δLIMs* (Fig. 1A). Within the *ßLIM* genes, two independent clades were found. One formed by dicotyledons, and another formed by monocotyledons. Although, no *ßLIM* genes were identified in barley (Fig. 1B).

Developmentally, *PLIM1* and *PLIM2* are relevant since the name derived from the preferential expression in pollen. Similarly, as *δLIM*, *PLIM1* genes were only found in lettuce, sunflower, and tobacco, which might suggest a unique and shared function between both subgroups. Unlike *PLIM1*, we identified *PLIM2* genes in dicotyledons and monocotyledons (Fig. 1B). Lettuce *PLIM2* have the same evolutionary origin than sunflower *PLIM2c* and *PLIM2d*. In monocotyledons, a clear irradiation was seen in all the species analyzed (Fig. 1A).

*DAR* genes are divided in three groups, *DAR-Class I*, *DAR-Class II*, and *DAR-like* genes. *DAR-Class I* and II genes were found in dicotyledons and monocotyledons. Interestingly, *DAR-like* was only found in *Arabidopsis* (Fig. 1B). We identified three lettuce *DAR-Class I*. *LsDAR1* and *LsDAR3* cladded with *AtDAR1*, *HaDAR5* and *HaDAR7*. *LsDAR4* formed a separated clade with *HaDAR2* (Fig. 1A). We also identified two DAR*-Class II* which cluster as expected with sunflower genes from the same subgroup. Monocotyledons *DAR-Class I* genes were more abundant than *DAR-Class II* and showed a strong conservation as only formed three clusters. Monocotyledons *DAR-Class II* have a common origin as all the members clustered together (Fig. 1A; Additional Fig. S1).

### Subcellular localization and physiochemical properties of LIM proteins

**Fig. 2:**
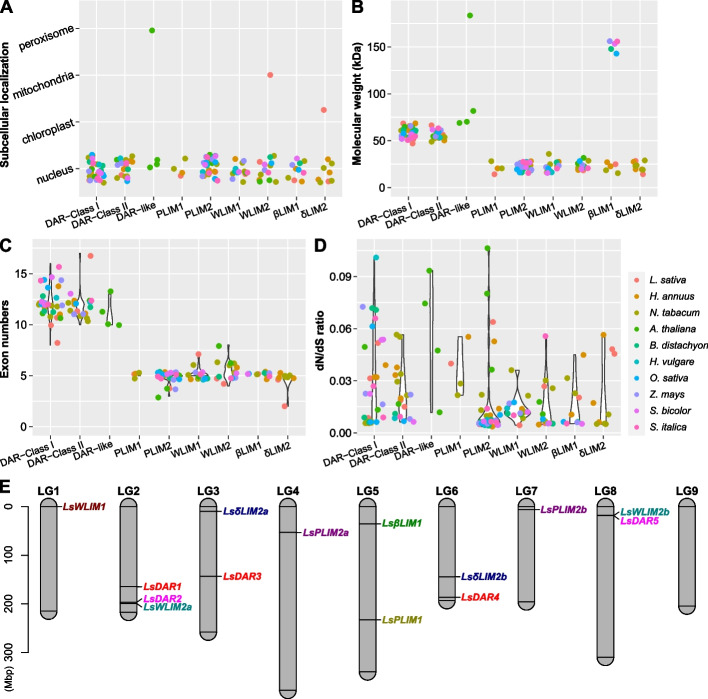
General characteristics of *LIM* genes of a set of dicotyledonous and monocotyledonous plant species. **A** Subcellular localization prediction of LIM proteins. **B** Molecular weight analysis of LIM proteins of a set of dicotyledonous and monocotyledonous plant species. **C** Exon number distribution of the LIM genes. **D** dN/dS ratios of LIM genes. **E** Mapping of lettuce *LIM* genes onto the linkage groups using the LinkageMapView package of *R* software. Genes were colored differently based on their classification

To further characterize the lettuce LIM family, we first predicted their subcellular localization (Fig. 2A) and collected their physiochemical characteristics including molecular weight (MW) (Fig. 2B), exon number (Fig. 2C), theoretical isoelectric point (pI), instability index, aliphatic index, chromosomal coordinates, and hydropathicity (Additional File 2: Table S2). Members of the lettuce LIM family showed similar MWs by subgroup (Fig. 2B). For instance, PLIM2, WLIM1 and WLIM2 average 20 kDa in weight. However, the DAR-related class average 50 kDa in weight in MWs ranging from 30 to 70 kDa (Fig. 2B). Interestingly, while PLIM2, WLIM1 and WLIM2 showed relatively similar number of exons, DAR-related genes showed a larger variability (Fig. 2C). It is noteworthy that even though LIM genes are described as transcription factors, a DAR-Like gene was predicted to be localized in the peroxisome. Similarly, a WLIM2 and a δLIM which were predicted to be localized in the mitochondria and chloroplast, respectively (Fig. 2A).

We then quantified the ratio of substitution rates at non-synonymous and synonymous sites in each *LIM* class of the dicotyledonous and monocotyledonous plant species analyzed to explore the evolutionary pressures on proteins. All the *LIM* classes in each of the dicotyledonous and monocotyledonous plants showed dN/dS ratio of lower that 0.10 (Fig. 2D). In general, no evidence of positive selection was found on any other member of the LIM genes based on the dN/dS ratio analysis (Fig. 2D; Additional File 3: Table S3), due to the fact that the hallmark signature of positive selection is accepted to be dN/dS > 1 [23].

### Distribution of LIM genes in the lettuce genome

Because the lettuce genome underwent whole-genome triplication [24], we explored the impact of this event on the distribution of the *LIM* gene family. *LIM* and *DAR* genes were randomly distributed in eight out of the nine lettuce chromosomes (Fig. 2E). No *LIM* genes were found in chromosome nine. Chromosome two has the largest number of *LIM* genes within a single chromosome with three members. Interestingly, no members of the same gene family are localized in the same chromosome but *LsDAR1* and *LsDAR2* in chromosome three. We did not find any duplication or triplication evidence in the lettuce *LIM* gene family (Fig. 2E).

### *LIM* genes share syntenic regions with dicotyledons and monocotyledons species

**Fig. 3:**
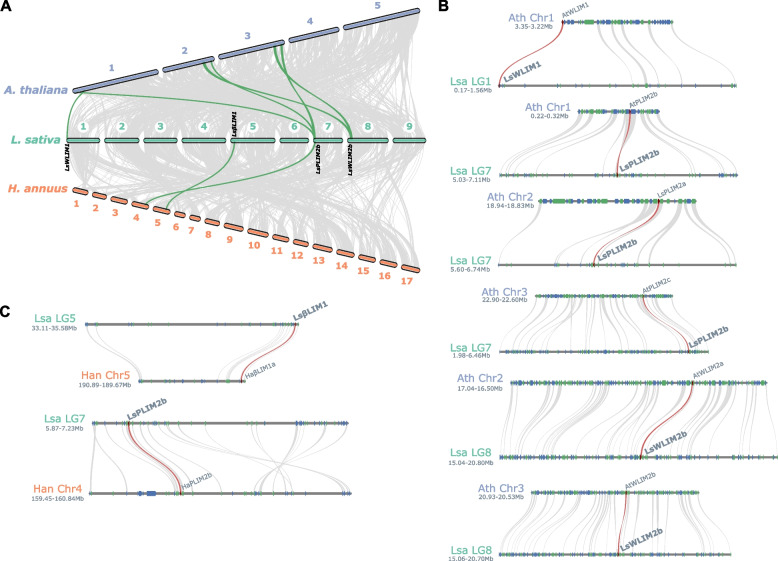
Syntenic analysis between lettuce *LIM* genes and other plant species. **A** Macrosynteny analysis of *LIM* genes in *A. thaliana*, *L. sativa*, and *H. annuus*. MCScan (python version) was utilized to identify syntenic pairs among *A. thaliana*, *L. sativa, and H. annuus* genomes. Gray lines represent all collinear blocks between the genomes of lettuce and other plant species, and green lines highlight syntelogs containing *LIM* genes. **B** Microsynteny analysis of *LIM* genes in *L. sativa* and *H. annuus*. Gray lines connect homologs in the collinear regions between lettuce and sunflower genomes, and red lines highlight *LIM* gene pairs*.* **C** Microsynteny analysis of *LIM* genes in *A. thaliana* and *L. sativa*. Gray lines connect homologs in the collinear regions between *Arabidopsis* and lettuce genomes, and red lines highlight *LIM* gene pairs

To further characterize the evolutionary relationship of *LIM* genes, we first constructed a comparative syntenic map using lettuce, sunflower and *Arabidopsis* (Fig. 3A). These three plant species share large syntenic regions in each of their chromosomes. Interestingly, we found more *LIM* genes in the syntenic regions shared with *Arabidopsis* than with sunflower (Fig. 3A). Our syntenic analysis showed that *LsßLIM1*, sited on chromosome 5, is syntenic to *HaßLIM1a* localized on chromosome 5 of sunflower. Interestingly, both *LsßLIM1* and *HaßLIM1a* are localized at the end of the chromosome likely closer to telomeric regions (Fig. 3B). The other syntenic *LIM* gene between sunflowers in lettuce is *PLIM2b*. While *LsPLIM2b* sits on chromosome seven, *HaPLIM2b* is localized on chromosome four (Fig. 3B). In *Arabidopsis*, all members of the *PLIM* subgroup, *AtPLIM1a*, *AtPLIM1b*, and *AtPLIM1c* share synteny with *LsPLIM2b* (Fig. 3C). Similarly, *LsWLIM1* and *LsWLIM2b* are syntenic to *AtWLIM1* and *AtWLIM2b*, respectively (Fig. 3C). We then expanded our syntenic analysis and found that lettuce shares LIM contained-syntenic regions with all monocotyledons and dicotyledons included in this study. However, the collinear regions of lettuce were larger with *Arabidopsis*, sunflower and tobacco compared to monocotyledon species (Additional Fig. S2).

### Gene structure of lettuce *LIM* genes

**Fig. 4:**
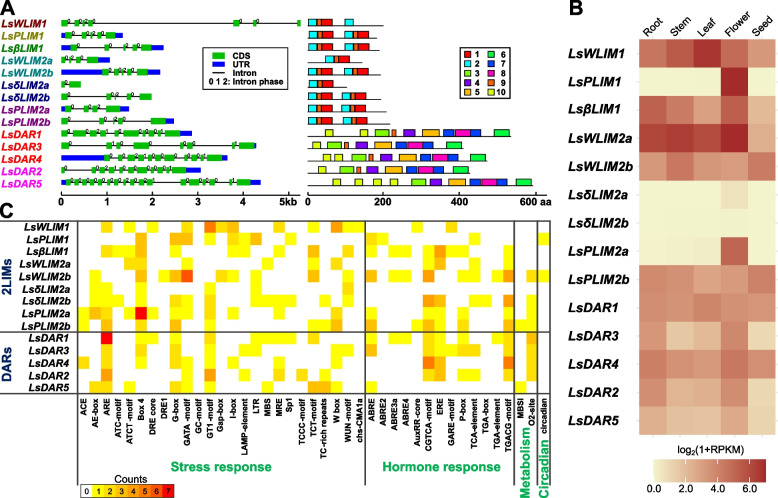
Structural analysis and developmental expression of *LsLIM* genes. **A** Exon–intron structures of lettuce *LIM* genes (the left panel) were drawn using Gene Structure Display Server (GSDS) v2.0. Blue boxes, green boxes, and black lines represent untranslated region (UTR), coding sequence (CDS), and intron, respectively. The numbers on each intron represent the intron phase (0, 1, or 2). Conserved motifs in the LIM protein sequences (the right panel) were discovered using the Multiple Em for Motif Elicitation (MEME) Suite tool. Lettuce LIM proteins with 10 identified motifs were shown. **B** Tissue expression (root, stem, leaf, flower, and seed) of lettuce *LIM* genes obtained from the LettuceGDB. **C** *Cis*-regulatory element analysis of promoter regions of LIM genes in lettuce. 2-kb upstream regions of *LsLIM* genes were analyzed to discover putative cis-regulatory elements using the PlantCARE tool. Four groups of *cis*-regulatory elements (stress response, hormone response, metabolism, and circadian rhythm) are displayed. The enrichment of each *cis*-regulatory element was represented using different colors. White, no occurrence; yellow to red, one to seven

*LIM* genes in lettuce have a variable number of exons and gene sizes (Fig. 4A). While *LIM* genes have a highly conserved gene structure, *DAR* genes have a more diverse motif composition and have larger proteins (Fig. 4A; Additional Fig. S3). Another interesting feature of the *DAR* genes compared to the LIM gene family is their variability in the intron phase. Unlike *2LIM* genes in lettuce which only contain phase 0 and phase 2 intron, *LsDAR* genes contain phase 1 introns in addition of phase 0 and 2 intron (Fig. 4A). Motif sequence information is available in Additional Fig. S3 and Additional File 4: Table S4.

### Developmental gene expression of lettuce *LIM* genes

To better understand the function of lettuce *LIM* genes, we examined their transcriptional expression in different tissues including roots, stems, leaves, flowers, and seeds using the transcriptome data of different tissues derived from LettuceGDB database [25] (Fig. 4B; Additional File 5: Table S5). Similarly, as reported in other plant species, most PLIM genes were expressed only in floral tissues. However, *LsPLIM2b* was ubiquitously expressed. Lettuce *WLIM* were expressed in all tissues analyzed as well as most of the *DAR* genes. Interestingly, lettuce *δLIM* genes did not show any expression of any tissue analyzed, while only *LsδLIM2a* shows a basal expression in flowers. The lack of expression of *δLIM* genes suggest a tissue specific expression and function in a different tissue than the ones explored in this study.

### *Cis*-regulatory element analysis of lettuce *LIM* gene promoters

In order to understand how gene expression of *LIM* genes is regulated, we performed a *cis*-regulatory element analysis of *LIM* gene promoters using a 2 kb upstream region of each gene. We particularly focused on *cis*-regulatory elements related to stress and hormone responses as well as metabolism and circadian rhythm (Fig. 4C; Additional File 6: Table S6). We identified 43 *cis*-regulatory elements among *LIM* gene promoters. Interestingly, within the stress responsive *cis*-elements, box 4, G-Box, GATA-motif, GT1 motif, ARE and w box were highly abundant in all *LIM* gene families. Interestingly, ARE motifs were found across the promoter of most *LIM* gene families except *WLIM2*. A similar enrichment of ARE motif was found on heat shock factor and heat shock proteins in lettuce [26], which suggest a role of this motif in the stress response. When we particularly looked at the *WLIMs* gene families, TCT-motif and box 4 were the most abundant in the stress responsive category (Fig. 4C). In the hormone stress category, CGTCA-motif, ERE, TGACG motif and ABRE were ubiquitously present in the LIM gene promoters. Interestingly, ABRE2, ABRE3a and ABRE4 motifs were underrepresented in promoter regions of most LIM genes. Within the *cis*-elements related to metabolism only O2-site was present across most *LIM* gene promoters. In contrast, MBSI was only present in three genes. Similarly, *cis*-elements related to circadian rhythm were overrepresented in all *LIM* gene promoters.

### Transcriptional expression analysis of LIM genes under different heat stress regimes

**Fig. 5:**
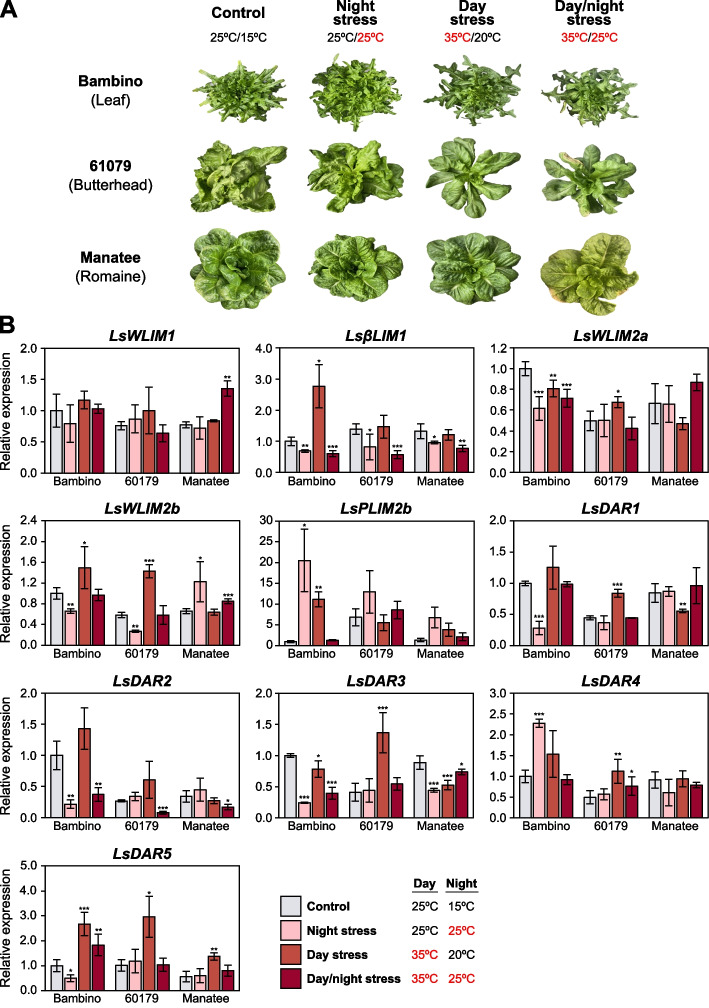
Transcriptional expression analysis of lettuce *LIM* genes under different heat stress regimes. **A** Phenotype of *L. sativa* cultivars Bambino (leaf), Manatee (romaine) and 60,179 (butterhead) at maturity after Control (25 °C/15 °C); night stress (25 °C/25 °C); day stress (35 °C/20 °C); day/night stress (35 °C/25 °C) treatments. **B** The relative expression of lettuce *LIM* genes. mRNA levels were determined by RT-qPCR analysis and normalized to *LsTUB* expression levels. Relative expression of treatment (night stress, day stress, and day/night stress) to the control are presented. Asterisks indicate significant differences between treatment and control determined by Student’s *t*-test (* *p* < 0.05, ** *p* < 0.01, *** *p* < 0.001)

To investigate the role of *LIM* genes under increased temperatures, we grew three different lettuce types, leaf (‘Bambino’), romaine (‘Manatee’) and butterhead (60,179) under different heat stress regimes. After germination, lettuce plants were placed into one of the following four temperature regimes: Control (25 °C/15 °C), night stress (25 °C/25 °C), day stress (35 °C/20 °C) and day/night stress (35 °C/25 °C). Light conditions were set at 450 ± 5 mmol·m^−2^·s^−1^ for an 11-h photoperiod, and CO_2_ levels were maintained at an optimum 400 ppm. The duration of each controlled heat treatment was dictated by the time from sow until the lettuce began to show preliminary signs of stem elongation (bolting) (Fig. 5A).

All tissue samples were collected before bolting began, at peak maturity, and used for RT-qPCR analysis to explore the transcriptional responses of lettuce *LIM* genes under different heat stress regimes. *LsWLIM1* did not show significant difference among the temperature regimes (Fig. 5B). *LsβLIM1* transcriptional expression changed in all three types of lettuce and in all temperature regimes analyzed. While *LsWLIM2a* expression was only significantly different in all temperature regimes in ‘Bambino’ and during day stress in 60,179, no differences were found in ‘Manatee’. *LsWLIM2b* expression changed in almost all temperature treatments. Interestingly, *LsWLIM2b* expression was downregulated during night stress in ‘Bambino’ and 60,179 but upregulated in ‘Manatee’ (Fig. 5B). *LsPLIM2b* was not expressed under control conditions, but all heat stress treatments upregulated its expression in all genotypes.

DAR genes also showed variability in gene expression after heat stresses. For instance, *LsDAR1* was significantly different during night stress in ‘Bambino’, and during day stress in 60,179 and ‘Manatee’ (Fig. 5B). *LsDAR2* showed downregulation in ‘Bambino’ during night and day/night stress. Interestingly, in 60,179 and ‘Manatee’ the low expression was only detected during combined day/night stress. *LsDAR2* also showed also mostly downregulation under all temperature regimes but upregulation during day stress in 60,179. *LsDAR4* was upregulated in ‘Bambino’ and 60,179 but not in ‘Manatee’ during night and day stress, respectively. *LsDAR5* was upregulated in ‘Bambino’, 60,179 and ‘Manatee’ during day stress. *LsPLIM1*, *LsPLIM2a*, *LSδLIM2a* and *LSδLIM2a* were not included in the analysis as their expression in leaves was not detected (Fig. 4B).

## Discussion

Long-term increased temperatures represent one of the most pressing threads for global food production as heat stress results in an overall decrease in plant growth and development and thus yield [2–6]. Current changes in human diets have positioned lettuce as one of the most highly consumed leafy vegetables. Therefore, breeding stress-tolerant lettuce cultivars able to withstand high temperature is one of the strategies needed to reduce the loss caused by abiotic and biotic stresses. Members of the *LIM* gene family have been identified in response to biotic and abiotic stressors [6, 17, 27, 28]. Lettuce proteins contain LIM domains with a unique double-zinc finger motif able to recognize DNA from downstream genes and thus good candidates for breeding purposes.

### Whole-genome duplication did not contribute to the diversification of *LIM* genes in dicotyledons and monocotyledons

Gene family expansion through gene duplication is a major driving force that generates gene diversification. In lettuce, a whole-genome triplication event occurred in lettuce since its divergence from the grape lineage [24]. While the increase in gene number in some gene families have been attributed to this event, others, for instance, the heat shock protein family 70 did increase due to whole-genome triplication but rather to independent tandem duplication events [26]. The identification of *LIM* genes has been mostly conducted in dicotyledonous. For instance, 15 members were found in 15 LIM in tomato (*Solanum lycopersicum L.) *[17]*,* twelve in poplar (*Populus trichocarpa*) [19] and 21 in alfalfa (*Medicago sativa L.)* [27]. Our syntenic analysis comparing lettuce to a set of dicotyledonous and monocotyledonous species reveals that, despite the presence of *LIM* genes in syntenic regions, their numbers did not undergo massive diversification as compared with other gene families [26], as shown by their number and chromosomal location (Fig. 1 and 2; Additional Fig. S2). These results indicate that *LIM* genes were not only conserved during the domestication of lettuce but are also highly conserved in angiosperms plants.

### *Cis-*elements composition and developmental specific expression

Cis-regulatory sequences located in promoters regulate gene expression and thus playing important roles in development and physiology. We identified that *LsLIM* genes contain many *cis*-elements related to stress responses (Fig. 4C). For instance, we identified anaerobic responsive elements (ARE), are bipartite elements consisting of GC and GT motifs, in the promoter regions of *LsPLIM* genes. AREs are essential for anaerobic induction [29]. Thus, it might be tentative to speculate that this *cis*-element may help in the resilience and resistance to decay under anaerobic conditions [30]. We also found the ABA responsive element (ABRE) in most of the *LsLIM* genes. Interestingly, we also identified ABRE2, ABRE3a and ABRE4. However, their expression was not widely distributed in *LsLIM* genes as the ABRE *cis*-element (Fig. 4C). The identification of *cis*-regulatory elements in vegetable crops open opportunities to study and engineer gene regulatory regions able to respond faster to changes in temperature and capable to maintain productivity under stressful conditions.

LIM genes are also strongly associated with their phylogenetic classifications. Three lettuce *LIM* genes among the fourteen identified in lettuce are only expressed in flowers (Fig. 4B). In some plant species, the *PLIM1* and *PLIM2* family genes are pollen specific and likely function in pollen growth and development. For instance, *Lilium longiflorum*, *LiLIM* play a role in pollen tube growth [31] and in *Nicotiana tabacum*, *NtPLIM1* function in pollen germination and pollen tube growth [32]. Similarly, in sunflower, *HaPLIM1a* is highly expressed in maturing pollen, suggesting its role in pollen tube growth and pollen germination [19]. The other ten genes were expressed across all the different tissues analyses which suggest the function in different developmental processes.

### Transcriptional response of *LIM* genes to increased temperatures

Transcription factors are fundamental for plant growth, development, and stress responses [13]. The ability of plants to respond to heat stress is strongly associated with the integration of gene regulators of more elaborated responses. Modulation of the transcriptional levels of transcription factor has been used as a strategy to regulate multiple downstream genes to improve stress tolerance [2, 33]. *LIM* genes have been identified in response to biotic and abiotic stresses in particular to heat stress [6], salinity, drought, and to the *Fusarium graminearum* infection [14, 17, 28]. We identified *LsLIM* genes that showed dynamic transcriptional expression under higher temperatures. Out of the fourteen identified *LsLIM* genes, ten belonging to the *WLIM, βLIM, PLIM, DAR* subgroups were responsive to at least one of the stress regimes imposed to lettuce plants during development (Fig. 5). Our transcriptional data show that *LsWLIM1*, *LsWLIM2b*, *LsDAR3* and *LsDAR5* participate in the heat stress response process to different heat stress regimes. In other plant species including tomato (*Solanum lycopersicum*) [17] and alfalfa (*Medicago sativa*) [27], exposure to high temperatures resulted in the activation of orthologous *LIM* genes indicating that these genes may have similar physiological functions. Noteworthy, it has been shown that *LIM* genes interact with b-ZIP transcription factors AREB1, AREB2, and ABF3 to jointly regulate plant response to abiotic stress [34]. Therefore, indicating that *LIM* genes might be part of conserved regulatory network involved in the stress response.

## Conclusions

Our detailed genome-wide analysis on the *LIM* gene family identified 14 genes in lettuce. Phylogenic analysis of *LIM* genes in monocotyledons and dicotyledons plant species highlight a close relationship with their orthologous *LIM* genes within these two clades. We identified reproductive specific members in several plant species as well as lettuce *LIM* genes responsive to increased temperatures. Our data show that *LsWLIM1*, *LsWLIM2b*, *LsDAR3* and *LsDAR5* as potential candidates for breeding programs aiming to produce lettuce varieties able to withstand higher temperatures under the current climate change scenario.

## Methods

### Plant material and growth conditions

Seeds from three different lettuce (*L. sativa*) types, leaf (‘Bambino’), romaine (‘Manatee’) and butterhead (60,179) were obtained from the lettuce breeding program at the University of Florida and pre-germinated for 48 h in a dark growth chamber until radicle emergence was observed [26]. Germinated seeds were transplanted into Pro-Mix® high-porosity (HP) Mycorrhizal soil and moved into light at room temperature. After 15 days following sowing, seedlings were transplanted into 3.78 L trade nursery pots prepared with 15 g 18–6-12 (N-P-K) osmocote and Pro-Mix® high-porosity (HP) Mycorrhizal soil inside a walk-in growth chamber using an average photosynthetic photon flux of 450 ± 5 mmol·m^−2^·s^−1^ with eleven-hour photoperiod (07:00 to 18:00).

### Heat stress regimes

Lettuce seedlings of similar size were used for each temperature regime. Plants from each cultivar and breeding line were grown for 15 days in the conditions previously described and then transferred inside growth chambers equipped with two multi-level shelves to one of the following heat stress regimes. Temperature regimes (day/night) were divided in 4 groups as follows: HT0: Control 25 °C/15 °C; HT1: 25 °C/25 °C; HT2: 35 °C/20 °C; HT3: 35 °C/25 °C, with day spanning from 07:00 to 18:00 and night from 18:01 to 06:59. Temperatures were gradually adjusted until they reached the set-up temperature. CO_2_ concentration and the relative humidity were set at 400 ppm and 60%, respectively. Each shelf contained plants distributed using a complete randomize block design.

### *LIM* sequences retrieval and Identification

The amino acid sequences of *LIM* genes in *A. thaliana* were collected from public database [19, 20, 35]. To identify *LIM* genes in other plant species (*B. distachyon*, *H. annuus*, *H. vulgare*, *L. sativa*, *N. tabacum*, *O. sativa*, *S. italica*, *S. bicolor*, and *Z. mays*), retrieved protein sequences were used as queries in BLASTP search against protein sequences of all nine plant species [24, 36–43] using 1·E^−10^ as an E-value threshold. The presence of the LIM domains (IPR001781) and DA1-like domain (IPR022087) was confirmed using InterProScan [44] and used as a criteria to retaining for further analyses.

### Phylogenetic Analysis and classification of *LIM* genes in plants

Once LIM genes were identified, multiple sequence alignment for 132 *LIM* genes of ten plant species were performed using the Multiple Sequence Comparison by Log-Expectation (MUSCLE) method [45]. The phylogenetic relationship among these genes was inferred by the Maximum Likelihood method based on Whelan and Goldman (WAG) model [21] with 1000 Bootstrap replications using MEGA11 software [46]. A discrete Gamma distribution was used to model evolutionary rate differences among sites [47]. Gene names and subgroups of LIM genes were assigned based on their phylogenetic relationship with previously reported genes (Additional File 1: Table S1).

### Physiochemical properties of *LIM* proteins

The subcellular localization of LIM proteins from ten plant species were estimated using WoLF PSORT online tool [48]. The physical and chemical parameters including molecular weight, theoretical isoelectric point, instability index, aliphatic index, and hydropathicity of all LIM proteins were computationally predicted using ProtParam tool in the ExPASy server [49].

### dN/dS ratio analysis

The protein sequence alignment between *LIM* genes from ten species and their corresponding *Marchantia polymorpha* homologs were performed using EMBOSS Water pairwise alignment in the EMBL-EBI data resources [50]. The non-synonymous to synonymous substitution ratio (dN/dS) was calculated based on the sequence alignment using the PAL2NAL server [51].

### Chromosomal mapping of LIM genes in lettuce

The chromosomal locations of *LIM* genes in the lettuce genome were based on annotation gff3 file, which was retrieved from Phytozome, *Lactuca sativa* V8 genome database [24, 52]. The *R* package LinkageMapView was used to depict the chromosomal distribution of lettuce *LIM* genes.

### Syntenic analysis of LIM genes

MCScanX tool was utilized to identify collinear blocks among plant genomes from *A. thaliana*, *B. distachyon*, *H. annuus*, *H. vulgare*, *L. sativa*, *N. tabacum*, *O. sativa*, *S. italica*, *S. bicolor*, and *Z. mays* [53]. Genome-wide syntenic relationship was displayed using the Circos tool [54]. Macro- and microsynteny visualization among *Arabidopsis*, lettuce, and sunflower genomes was conducted using MCScan (python version) from jcvi [55].

### Gene structure, motif, and *cis*-regulatory element analysis

Exon–intron organization of lettuce *LIM* genes were illustrated using Gene Structure Display Server (GSDS) v2.0 [56]. Multiple Em for Motif Elicitation (MEME) Suite online tool was used to identify conserved motifs in LIM proteins across ten plant species [57]. The following parameters were used for MEME motif discovery: discovery mode = classic, number of repetitions = any, number of motifs = 10, optimum motif length = 6 to 50 residues. *Cis*-regulatory elements in the 2-kb upstream regions of *LsLIM* genes were discovered using the PlantCARE database [58].

### Expression analysis of lettuce LIM genes

To analyze the expression patterns of lettuce *LIM* genes, we obtained RNA-seq data from the lettuce genome across different tissue types from LettuceGDB [25]. The Reads Per Kilobase Million (RPKM) values were transformed by log_2_(1 + RPKM). The heatmap of each *LsLIM* gene was visualized using R software.

### RNA isolation and RT-qPCR analysis

Total RNA was extracted using Trizol® reagent (Invitrogen) according to the manufacturer’s instruction. DNase treatment was performed using DNase I (Thermo Scientific). First-strand cDNA synthesis was conducted using iScript™ cDNA Synthesis Kit (Bio-Rad). Quantitative real-time PCR was performed using AzuraView™ GreenFast qPCR Blue Mix LR reagent (Azura Genomics) in the QuantStudio™ 3 Real-Time PCR System (Applied Biosystems) on a 96-well reaction plate. Reaction conditions consisted of pre-denaturation step at 95 °C for 5 min and 45 cycles of 95 °C for 15 s, 60 °C for 30 s, and 72 °C for 30 s. The melting curve analysis was followed using 95 °C for 15 s, 60 °C for 60 s, and 95 °C for 15 s [59]. Relative transcript levels were normalized using *LsTUB* gene [26] and calculated based on 2^−ΔΔCt^ method [60]. Student’s *t*-test was used to assess the significant differences between control condition and stress regime within the same genotypes. Primers used in RT-qPCR analysis are listed in Additional Table 7: Table S7.

### Statistical analysis

R software/environment was used for the statistical analyses of the RT-qPCR data. We used data from three independent experiments. Three biological replicates with three technical repetitions per biological replicate were used. Student’s t-test was used to compare gene expression among the different heat stress regimes and the control conditions. Differences in means were considered significant at p-value < 0.05.

### Supplementary Information

Additional file 1: Table S1. List of previously reported *LIM* genes in different plant species.

Additional file 2: Table S2. List of *LIM* genes and their physiochemical characteristics.

Additional file 3: Table S3. dN/dS ratio of *LIM* genes in different plant species.

Additional file 4: Table S4. Detailed information of conserved motifs identified by MEME analysis.

Additional file 5: Table S5. Expression profiles of lettuce *LIM* genes in different tissues.

Additional file 6: Table S6. *Cis*-regulatory element analysis of 2-kb upstream regions of lettuce *LIM* genes.

Additional file 7: Table S7. Primers used in this study.

Additional file 8: Supplemental Figure S1. Phylogenetic analysis of *LIM* genes from ten plant species. The protein sequences from *A. thaliana* (At), *B. distachyon* (Bd), *H. annuus* (Ha), *H. vulgare* (Hv), *L. sativa* (Ls), *N. tabacum* (Nt), *O. sativa* (Os), *S. italica* (Si), *S. bicolor* (Sb), and *Z. mays* (Zm) were utilized for multiple sequence alignment and phylogenetic tree construction. Phylogenetic relationship was inferred using the Maximum Likelihood method, employing Whelan and Goldman (WAG) model and a discrete Gamma distribution. The numbers at the nodes represent the percentage of bootstrap values, based on 1000 replications. Additional Figure S2. Syntenic analysis of *LIM* genes of ten plant species. Syntelog anchors among different plant genomes of *A. thaliana* (Ath), *B. distachyon* (Bdi), *H. annuus* (Han), *H. vulgare* (Hvu), *L. sativa* (Lsa), *N. tabacum* (Nta), *O. sativa* (Osa), *S. italica* (Sit), *S. bicolor* (Sbi), and *Z. mays* (Zma) were identified using MCScanX [53]. The syntenic relationships among genomes were depicted using Circos [54]. Gray, orange, and red lines indicate all syntelogs, syntelogs among LIM genes, and *LsLIM*-containing syntelogs, respectively. Additional Figure S3. Conserved motifs of LIM proteins discovered using MEME analysis.

## Data Availability

All data generated or analyzed during this study are included in this published article and its supplementary information files.
