# Supplementary figures and images for: Global identification of LIM genes in response to different heat stress regimes in Lactuca sativa
Source: BMC Plant Biol. 2024 Aug 6;24:751. doi: 10.1186/s12870-024-05466-x (PMC11301969; doi:10.1186/s12870-024-05466-x)

Supplemental Figure S1

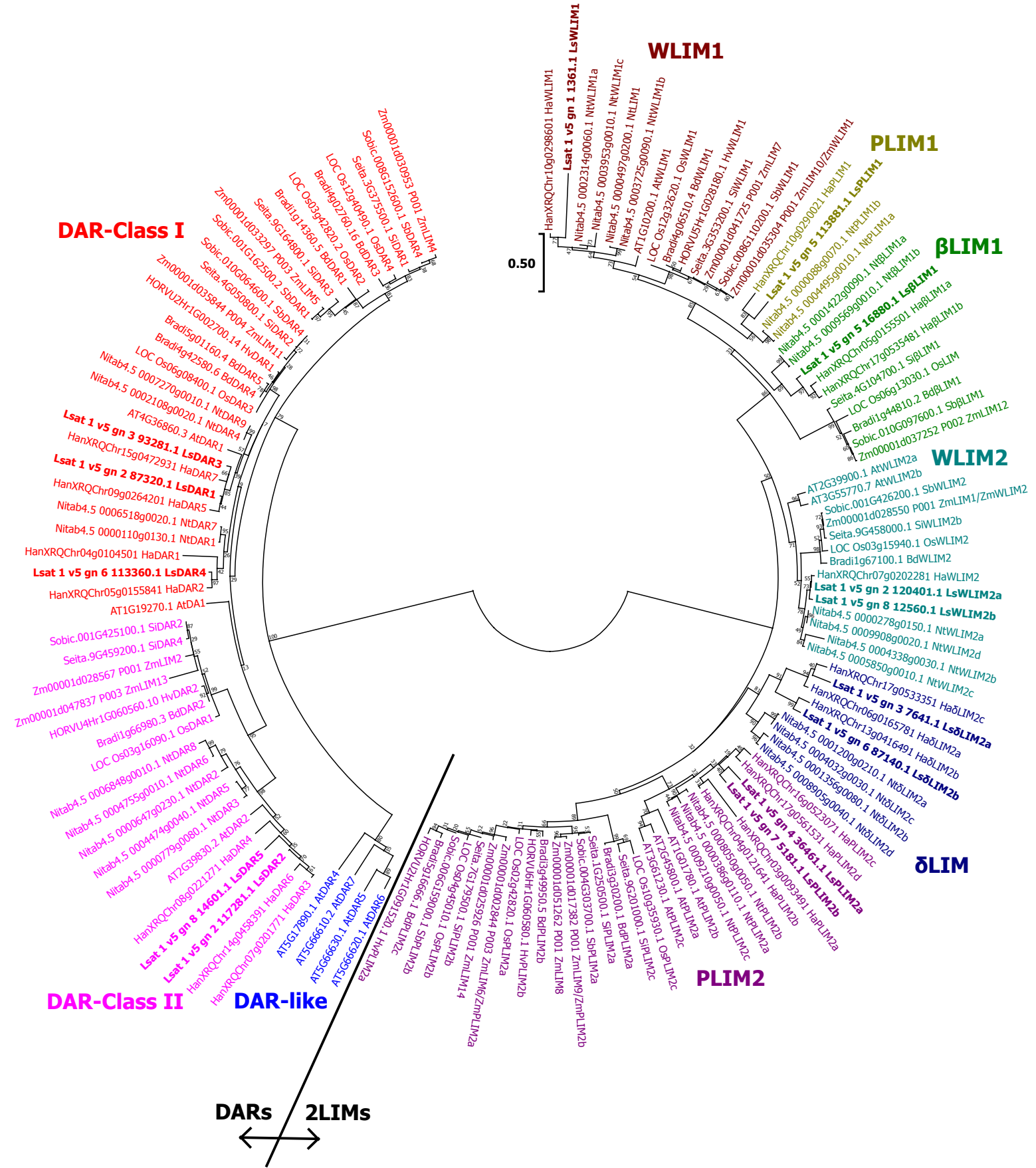

Supplemental Figure S2

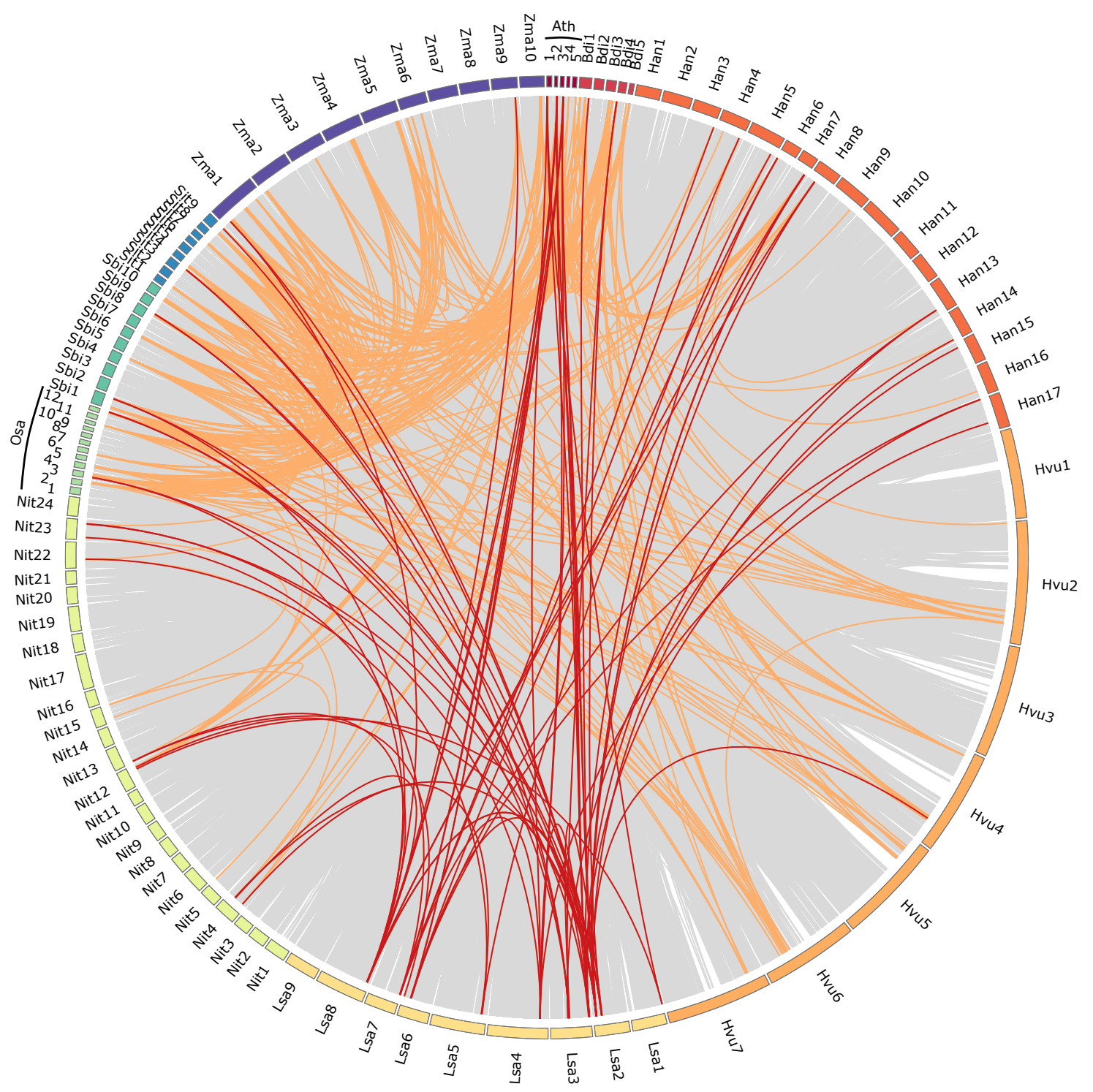

### Supplemental Figure S3

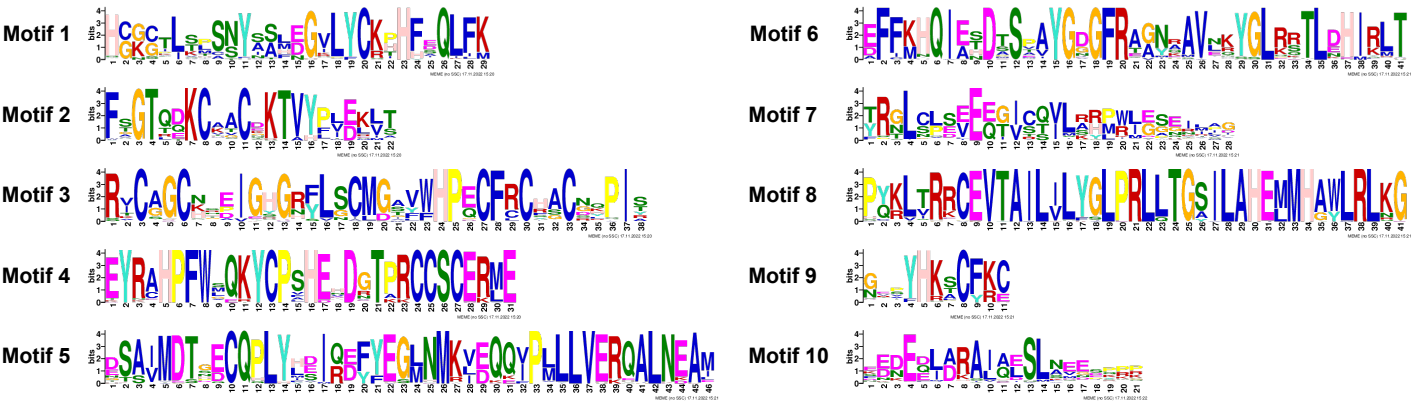

Supplement: Supplementary file 8 — Additional file 8: Supplemental Figure S1. Phylogenetic analysis of LIM genes from ten plant species. The protein sequences from A. thaliana (At), B. distachyon (Bd), H. annuus (Ha), H. vulgare (Hv), L. sativa (Ls), N. tabacum (Nt), O. sativa (Os), S. italica (Si), S. bicolor (Sb), and Z. mays (Zm) were utilized for multiple sequence alignment and phylogenetic tree construction. Phylogenetic relationship was inferred using the Maximum Likelihood method, employing Whelan and Goldman (WAG) model and a discrete Gamma distribution. The numbers at the nodes represent the percentage of bootstrap values, based on 1000 replications. Additional Figure S2. Syntenic analysis of LIM genes of ten plant species. Syntelog anchors among different plant genomes of A. thaliana (Ath), B. distachyon (Bdi), H. annuus (Han), H. vulgare (Hvu), L. sativa (Lsa), N. tabacum (Nta), O. sativa (Osa), S. italica (Sit), S. bicolor (Sbi), and Z. mays (Zma) were identified using MCScanX [53]. The syntenic relationships among genomes were depicted using Circos [54]. Gray, orange, and red lines indicate all syntelogs, syntelogs among LIM genes, and LsLIM-containing syntelogs, respectively. Additional Figure S3. Conserved motifs of LIM proteins discovered using MEME analysis. [file 12870_2024_5466_MOESM8_ESM.pdf]
